# Comprehensive analysis of cellular metrics: From proliferation to mitochondrial membrane potential and cell death in a single sample

**DOI:** 10.1038/s41420-025-02391-2

**Published:** 2025-03-24

**Authors:** Sirina Sabirova, Gulnaz Sharapova, Aida Budyukova, Marina Gomzikova, Anna Brichkina, Nick A. Barlev, Albert Rizvanov, Nikita Markov, Hans-Uwe Simon

**Affiliations:** 1 Laboratory of Molecular Immunology, Institute of Fundamental Medicine and Biology, Kazan Federal University, Kazan, Russia; 2 Laboratory of Intercellular Communication, Kazan Federal University, Kazan, Russia; 3 OpenLab Gene and Cell Technology, Institute of Fundamental Medicine and Biology, Kazan Federal University, Kazan, Russia; 4 Institute of Systems Immunology, Center for Tumor Biology and Immunology, Philipps University of Marburg, Marburg, Germany; 5 Institute of Cytology of the Russian Academy of Sciences, St. Petersburg, Russia; 6 Department of Biomedical Sciences, School of Medicine, Nazarbayev University, Astana, Kazakhstan; 7 Division of Medical and Biological Sciences, Tatarstan Academy of Sciences, Kazan, Russia; 8 Institute of Pharmacology, University of Bern, Bern, Switzerland; 9 Institute of Biochemistry, Brandenburg Medical School, Neuruppin, Germany

**Keywords:** Cell death, Cell division

## Abstract

Changes in cell number during in vitro experiments and pharmacological screenings primarily depend on two factors: cell death and proliferation. The dynamics of these processes determine whether cell populations expand and accumulate or, conversely, decrease over time. Understanding the biological mechanisms governing these changes is crucial for deciphering the mode of action of any pharmacological or genetic treatment in fundamental research and pre-clinical trials. In this context, we introduce a robust and efficient flow cytometry-based methodology that enables comprehensive analysis of key cellular parameters that indicate changes in cell numbers. This approach encompasses the assessment of cell count along with critical maintenance parameters including proliferation, cell cycle dynamics, apoptosis, cell permeability, and mitochondrial depolarization. These parameters are intricately linked, offering a detailed view of the cellular state. The described methodology is versatile and adaptable for analyzing various cell types, whether at steady state or in response to treatments. To develop this workflow, we integrated and optimised multiple flow cytometry-based stainings such as annexin V, propidium iodide, bromodeoxyuridine, CellTrace Violet, and JC-1 into a unified protocol. This article offers a detailed, step-by-step guide to the entire method, covering aspects such as timing, sample preparation techniques, and the reagents used. Additionally, it includes examples of the data that can be obtained with this technique and illustrates its multiparametric visualization. Collectively, this methodology facilitates the rapid acquisition of up to eight different parameters from a single sample in one experiment.

## Introduction

Flow cytometry is a technique that enables the simultaneous detection of cells and objects varying in size and granularity, as well as the quantification of fluorescent signals emitted by different stains and conjugated antibodies. As a multiparametric method, it is capable of characterizing a wide array of cellular processes, including cell cycle dynamics, proliferation rates, apoptosis, autophagy, ion homeostasis, organelle functions, and immunophenotyping [1–3]. Moreover, its widespread usage is attributed to its capability to individually measure fluorescent signals in each cell at rates up to 70,000 events per second [3]. Unlike fluorescent and confocal microscopy, flow cytometry is less susceptible to background signal fluctuations and eliminates the bias associated with selecting specific regions on microscopy slides for analysis. Typically, flow cytometry analyses a minimum of 10,000 cells per experiment, significantly surpassing the few hundred or thousands of cells typically assessed by microscopy methods. Additionally, most flow cytometry instruments automatically calculate signal intensities, presenting results as mean, geometric mean, or median values, which eliminates the necessity for additional software and extensive post-acquisition analysis commonly required in microscopy.

**Table 1:** Stock reagents.

Reagent	Fluorochrome Ex/Em	Source	Catalog #	Weight	Volume	Stock [c]
BrdU	-	Sigma-Aldrich St. Louis, Missouri, United States	59-14-3	9.2 mg	1.498 ml DMSO	20 mM
Alexa Fluor® 647 Mouse anti-BrdU antibody	650/671 (filter for APC)	BD Franklin Lakes, New Jersey, United States	560209	–	–	–
Propidium iodide	535/617 (filter for PE)	Thermo Fisher Scientific Waltham, Massachusetts, United States	P3566	–	–	1 mg/ml (1.5 mM)
CellTrace™ Violet	405⁄450 (filter for BV421)	Thermo Fisher Scientific Waltham, Massachusetts, United States	C34557	-	50 μl DMSO	2 mM
JC-1	514/529 514/590 (filters for FITC and PE)	Enzo Life Sciences Farmingdale, New York, United States	ENZ-52304	5 mg	1 ml DMSO	7.66 mM
Annexin V binding buffer	–	–	–	–	–	10x
APC Annexin V	651/660 (filter for APC)	BD Franklin Lakes, New Jersey, United States	550474	–	–	–
RNase A	–	Sigma-Aldrich St. Louis, Missouri, United States	R4875	10 mg	1 ml PBS	10 mg/ml
Borax Anhydrous (Sodium teraborate)	–	Sigma-Aldrich St. Louis, Missouri, United States	71997-100 G	11.4 g	300 ml ddH_2_O	100 mM pH 8.5
Tween-20	–	Sigma-Aldrich St. Louis, Missouri, United States	8221841000	–	–	100%
Triton X-100	–	Sigma-Aldrich St. Louis, Missouri, United States	1086031000	–	–	100%
12-well Cell Culture Plate (or equivalent)	–	Fisher Scientific Hampton, New Hampshire, United States	07-000-208	–	–	–
DMEM, high glucose, GlutaMAX™ Supplement, pyruvate	–	Thermo Fisher Scientific Waltham, Massachusetts, United States	31966021	–	–	–
Trypsin-EDTA (0.25%), phenol red	–	Thermo Fisher Scientific Waltham, Massachusetts, United States	25200072	–	–	–

List of reagents required for protocol implementation.

**Table 2:** Equipment.

Equipment	Source
HERAcell 150 CO^2^ Incubator	Thermo Fisher Scientific Waltham, Massachusetts, United States
BD FACSLyric flow cytometer	BD Franklin Lakes, New Jersey, United States
Microcentrifuge MIKRO 220 R	Hettich Tuttlingen, Germany
BioSan TDB-120 solid state thermostat	BioSan, Riga, Latvia

Equipment used in this study. Generally, any instrumentation with similar specifications can be used to implement the protocol.

**Table 3:** Final dilutions.

Reagent	Stock [c]	Dilution from the stock solution	Working [c]
CellTrace Violet	2 mM	1:666,7	3 µM
BrdU	20 mM	1:1000	20 µM
Anti-BrdU antibody	–	1:50	1 µl per test
RNAse A	10 mg/ml	1:1000	10 µg/ml
Propidium Iodide	1.5 mM	1:500	3 µM
JC-1	7.659 mM	1:2553	3 µM
Annexin V	–	1:25	2 µl per test

Working concentrations of the reagents used.

In this study, we introduce a single protocol that integrates multiple flow cytometry assays designed to elucidate the biological reasons behind changes in cell numbers, primarily governed by cell death and cell proliferation. These factors, in turn, are influenced by a range of biological processes, including apoptosis, mitochondrial dynamics, and cell cycle progression [4, 5]. For example, mitochondrial depolarization can trigger the release of cytochrome c, thereby initiating the intrinsic apoptosis pathway [6]. Additionally, even mild mitochondrial depolarization can impair energy production, which may reduce cell proliferation rates and render cells more vulnerable to various treatments. Moreover, the dynamics of cell cycle progression directly regulate proliferation rates and provides insight on specific features, including cell cycle arrest linked to prolonged DNA synthesis in the S phase, inhibition of division in the G2 phase, or the induction of senescence in the G1 phase [7]. The methodology we propose here enables the detection of all these parameters from a single sample of approximately half a million cells within approximately 5 h.

**Fig. 1:**
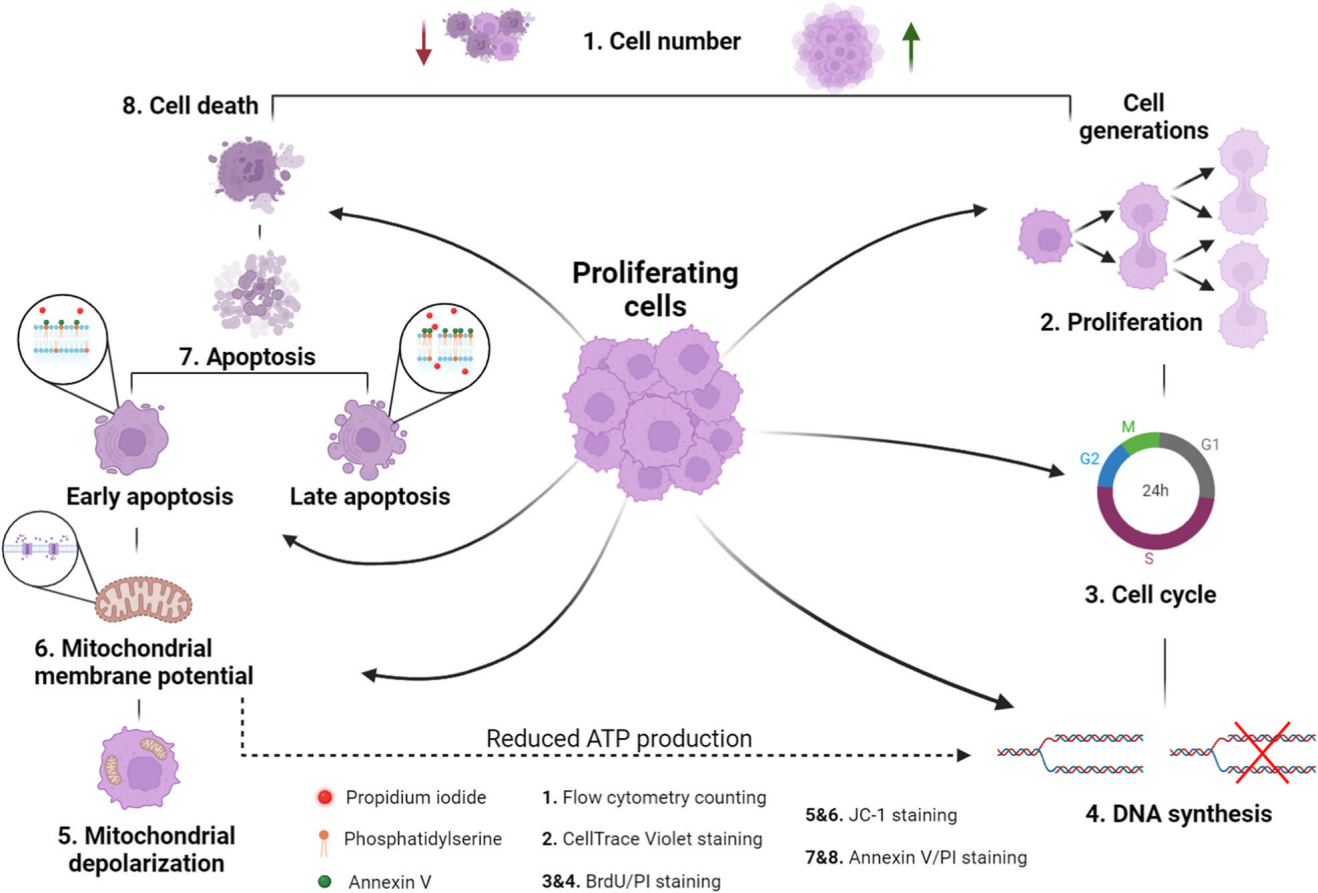
Changes in cell number are governed by alterations in proliferation and cell death. Changes in cell number are governed by alterations in proliferation and cell death. Cell death is most commonly associated with apoptosis, which progresses through early and late stages, distinguished by the intact or permeable status of cell membranes, respectively. Apoptosis is often triggered by mitochondrial depolarization and the subsequent release of cytochrome c. Thus, mitochondrial depolarization is linked to both the induction of apoptosis and a reduction in ATP production, as mitochondrial energy production relies on the mitochondrial membrane potential. Conversely, cell proliferation drives increases in cell numbers. Proliferation is characterized by the rate at which cells progress through the stages of the cell cycle, culminating in mitosis. Cells often spend a considerable amount of time in the S phase, and the rate of DNA synthesis during this phase can provide insights into the overall cellular status and energy balance. The protocol described in this paper integrates multiple flow cytometry stainings, enabling the evaluation of all these parameters.

In this protocol, we utilized well-established staining techniques such as BrdU (bromodeoxyuridine), PI (propidium iodide), annexin V, JC-1, and CellTrace Violet (a CFSE-like dye). BrdU and PI together are used to monitor cell cycle dynamics, JC-1 measures mitochondrial membrane potential, Annexin V/PI staining assesses cellular death through the permeabilization of the cellular membrane and apoptosis, and CellTrace Violet dye is employed to measure proliferation rates and trace cell generations. This combination of stainings enables the collection of comprehensive data on DNA synthesis intensity, cell cycle status, mitochondrial membrane potential, the number of cells with depolarized mitochondria, and levels of early and late apoptosis. Collectively, the simultaneous analysis of these parameters provides a comprehensive overview of cellular status and fate, offering a deep understanding of the mechanisms driving changes in cell numbers (Fig. 1).

## Discussion

The decrease in cell number can be attributed to either diminished cell proliferation or increased cell death. The workflow outlined here enables a comprehensive understanding of how changes in cell numbers generally occur and distinctly identifies whether decreased proliferation or increased cell death is the main factor influencing the outcome under any given treatment. Additionally, this methodology reveals whether changes in mitochondrial depolarization and apoptosis are the underlying causes of observed alterations in cell death, or if modifications in the cell cycle, including cell cycle arrest, are responsible for shifts in proliferation dynamics.

Given that many of these changes are often interconnected, and that it is common to observe coherent shifts in these parameters following various treatments or stimuli, this analysis provides multilevel evidence that supports the hypothesized mechanism. This approach ensures that the observed changes are not isolated phenomena but are part of a broader, interconnected response to the treatments applied.

In this workflow, we did not concentrate on two additional aspects and data points that could be derived from these staining techniques. Specifically, CellTrace Violet and similar CFSE-like dyes do more than just calculate the relative proliferation rate; they also enable the assessment of the number of cell generations produced within the examined period and the proportion of cells that are capable or incapable (senescent) of proliferation [8, 9]. We omitted the focus on these features because they are generally not common for cancer cell lines, which are immortal and continuously proliferating, typically devoid of senescent cells or distinct generations. However, it is crucial to note that these features can also be characterized using this staining technique and may be particularly significant in certain contexts, such as tracing T cell generations or evaluating senescent cells in projects related to aging.

Similarly, BrdU incorporation can be utilized to ascertain not only the percentage of cells in different phases of the cell cycle but also the intensity of DNA synthesis during the S phase. The intensity of the BrdU signal is indicative of the amount of BrdU integrated into DNA during replication. This integration rate is influenced by the speed of DNA synthesis, which reflects how quickly cells progress through the S phase. Factors such as overall cell stress, ATP availability, and other cellular conditions significantly impact this process. Therefore, the intensity of BrdU incorporation can serve as a proxy for assessing the overall cellular status and homeostasis.

This protocol offers potential for expansion based on the specific objectives of researchers. For example, incorporating caspase-specific fluorescent probes would enable the detection of early stages of apoptosis initiation [10, 11]. This approach is often more sensitive and capable of detecting subtle changes in apoptosis dynamics compared to annexin V, which is more commonly associated with the terminal stages of apoptosis. Similarly, the inclusion of γH2AX staining can be used to assess the level of DNA damage [12]. Additionally, dyes such as dihydrorhodamine (DHR) or dichlorodihydrofluorescein diacetate (DCFDA) can be employed to measure levels of reactive oxygen species which are closely linked to oxidative stress levels [13]. These expansions could provide a more comprehensive understanding of cellular responses under various conditions, potentially enhancing the utility of this protocol in biomedical research. Interestingly, our data on the selective effects of the ETC inhibitors both align with and expand on a study reported on K562 leukemia cells, where the Complex III inhibitor antimycin A also caused a pronounced reduction in G1-phase cells and accumulation in the S phase, a phenomenon linked to disrupted purine synthesis and imbalanced NADP/NADPH ratios [14]. In the CRC cell lines only antimycin A, and not the Complex I inhibitor rotenone, triggered a shift to the S phase. This indicates that while both compounds induce dysfunction of the ETC and subsequent ATP depletion, only Complex III inhibition and the following metabolic changes exert a sufficient effect on de novo nucleotide biosynthesis and redox homeostasis, ultimately leading to cell cycle arrest.

## Method

### Staining principles

#### BrdU/PI staining

BrdU/PI staining is a technique utilized to assess cell cycle progression and proliferation [15, 16]. The cell cycle consists of four primary phases: G1 (presynthetic), S (synthetic), G2 (postsynthetic), and M (mitotic), with the mitotic phase culminating in the division of the cytoplasm and plasma membrane [17]. BrdU, a thymidine analog, is used to monitor these changes [18]. During DNA replication, BrdU is incorporated into the DNA, marking cells in the S phase. Concurrently, cells are stained with PI, which binds to nucleic acids. The intensity of PI fluorescence correlates with the DNA content, facilitating the identification of cells in the G1 phase (single chromosome set) and G2 phase (double chromosome set). Thus, BrdU/PI staining effectively determines the proportion of cells in each significant phase of the cell cycle: G1, S, and G2.

#### Annexin V/PI staining

Annexin V/PI staining is utilized to detect apoptotic, live, and dead cells, offering a distinct advantage in differentiating between cells undergoing various apoptotic or necrotic forms of death [19]. Annexin V, a calcium-binding protein, is widely used for apoptosis detection due to its high affinity for phosphatidylserine (PS) on the plasma membrane. In healthy cells, PS is typically confined to the inner leaflet of the plasma membrane and does not interact with annexin V, which binds only to the outer side. However, in apoptotic cells, PS is externalized to the outer membrane, making it accessible for annexin V binding [19–21]. Staining cells with fluorescently labeled annexin V enables the identification of apoptotic cells via flow cytometry. Propidium iodide, on the other hand, penetrates cells only when the membrane integrity is compromised, becoming permeable to small molecules, thus marking cell death. PI is typically used alongside annexin V to detect dying cells that exhibit both membrane permeability and externalized PS. In our analysis, cells that were permeable to PI but did not bind annexin V were classified as necrotic, following common literature practices. Collectively, this technique allows for the distinction of four cell groups: healthy cells (PI−/Annexin V−), early apoptotic cells (PI−/Annexin V+), dying cells in the late stages of apoptosis with permeable membranes (PI+/Annexin V+), and necrotic cells (PI+/Annexin V−).

#### JC-1 staining

Mitochondrial status is tightly connected to cell death and proliferation owing to the direct involvement of these organelles in the induction of apoptosis and energy production [6, 22]. To assess the mitochondrial status of cells, our protocol incorporates JC-1 staining (5,5′,6,6′-Tetrachloro-1,1′,3,3′-tetraethyl-imidacarbocyanine iodide). JC-1 dye allows the quantification of depolarization or hyperpolarization of the mitochondrial membrane [23]. JC-1 dye is particularly useful for quantifying changes in the mitochondrial membrane potential (MMP) as it exhibits dual fluorescence properties. In its monomeric form, JC-1 emits green fluorescence, but within active mitochondria, it aggregates into structures that emit red fluorescence known as J-aggregates [24]. This dual behavior allows JC-1 to differentiate between hyperpolarized and depolarized mitochondrial states. The ratio of red to green fluorescence directly corresponds to the MMP; a higher red/green ratio indicates a higher MMP. The ratiometric nature of JC-1 staining not only distinguishes between normal and reduced MMP but also provides more precise quantitative measurements compared to non-ratiometric dyes like TMRE. This capability is further enhanced by flow cytometry, which facilitates the gating of cells based on their MMP status. By correlating the data gathered from Annexin V/PI and JC-1 staining, it is possible to determine whether cells are experiencing mitochondrial depolarization accompanied by the induction of apoptosis, or if the depolarization does not influence cell death but merely restricts energy production and cell proliferation. This approach allows for a nuanced understanding of the relationship between mitochondrial function and cell fate.

#### CellTrace Violet staining

CellTrace Violet dye, a CFSE-like dye, is used for in vitro and in vivo labeling of cells to trace their generations using the stoichiometric dye dilution principle and flow cytometry acquisition [25]. This dye freely penetrates the cytoplasmic membrane and covalently binds to all free amines. At low micromolar concentrations (up to 5 µM) CellTrace Violet does not display cytotoxicity. During cell division, the dye is evenly distributed among the daughter cells and the fluorescence intensity is decreased by half. Due to its ability to retain in cells for long intervals of time without leaking into neighboring cells, CellTrace Violet dye allows tracing generations of proliferating cells as well as detecting senescent or non-dividing cells. The signal of CellTrace Violet and similar dyes is usually detected by flow cytometry, which visualizes individual cell generations until the dye's fluorescence reaches inherent cell autofluorescence levels. By dividing the signal intensity obtained at the start of the experiment by that at the end, it is possible to determine the relative proliferation rate of the cells, considering that dye loss is solely attributed to the number of cell divisions. Consequently, cells that have divided once exhibit a twofold reduction in staining, those that have divided twice show a fourfold reduction, and those that have divided three times display an eightfold reduction in signal.

#### Quantification of cell numbers

Additionally, this protocol utilizes cell counting using time-dependent flow cytometry acquisition of events. Modern flow cytometry instruments allow acquiring events in a time-dependent fashion with respect to their size and granularity. For instance, the flow cytometer BD FACSLyric, acquires 60 µl/min in the medium speed mode, allowing straightforward calculation of cell concentration and cell number in the original sample. Moreover, flow cytometry-based time-dependent measurements of cells allow quantification of cell numbers and differentiation between normal cells and debris or apoptotic bodies [26]. Although cell counts obtained in this way may vary slightly compared to those derived from normalization using cell counting beads, this method remains sufficiently accurate for determining relative changes in cell numbers across different conditions, assuming that the same analytical errors apply uniformly to all tested samples.

#### Protocol overview

**Fig. 2:**
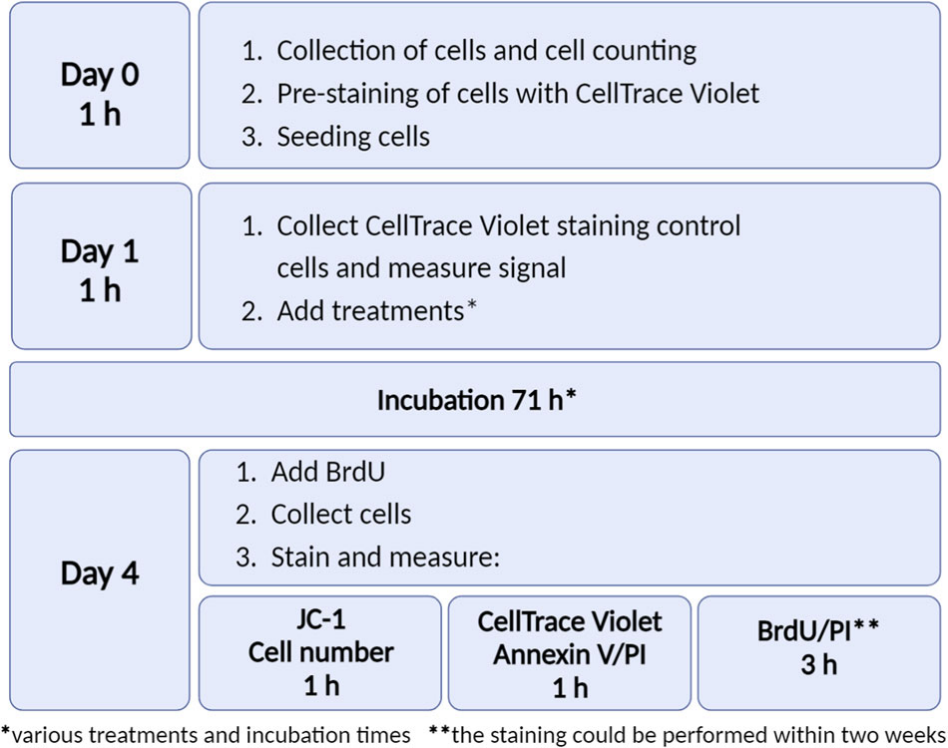
The overall timeframe of the analysis. *Incubation 72 h (71 h treatment incubation + 1 h BrdU incubation): The incubation time is flexible but typically should not exceed 96 h for two main reasons: (1) most commonly used cell lines may approach confluence after 96 h when seeded at a reasonable initial concentration; (2) beyond 96 h, due to cell proliferation, most of the CellTrace Violet dye is likely to be lost when used at 5 μM concentration, and the remaining signal may approach the levels of autofluorescence. **After the initial ethanol fixation step in the protocol, cells can be stored at +4 °C for up to two weeks directly in tubes, allowing for delayed processing without immediate analysis.

Overall, the protocol outlined involves parallel/sequential detection through four staining techniques (BrdU/PI, CellTrace Violet, Annexin V/PI, JC-1) alongside cell number counting, executed as three distinct flow cytometry measurements. These measurements collectively quantify eight parameters: cell number, proliferation rate, cell cycle status and DNA synthesis intensity, early and late apoptosis, death-associated cell permeability, the presence of cells with depolarized mitochondria, and mitochondrial membrane potential. This protocol has been refined to generate a large volume of robust and reliable data with relatively low effort. The total time required for sample preparation and subsequent analysis using the flow cytometer is approximately 5 h (Fig. 2).

### Before you begin

#### Preparation of stock reagents, examples of required equipment and final dilutions

See Tables 1–3.

##### General preparations for flow cytometry

During pilot experiments, properly set up the flow cytometer by selecting the appropriate filters for each dye or fluorophore used. Adjust the PMT (photomultiplier tube) voltages to ensure that the fluorescence emitted falls within the dynamic range of the instrument. This step is critical to achieving accurate and reliable fluorescence measurements. To replicate the current protocol, you need to have access to a flow cytometer equipped with three lasers (violet—405 nm, blue—488 nm and red—640 nm). For data acquisition, we suggest creating of three templates:

- *Template 1:* for JC-1 staining and cell counting:
  JC-1 is excited by a 488 nm laser. Use filters corresponding to the detection of FITC and PE fluorophores for the detection of green and red signals, respectively. Acquire for 40 s.
- *Template 2:* for CellTrace Violet staining and annexin V/PI staining:
  CellTrace Violet is excited by a 400 nm laser and detected by a filter corresponding to detection of BV421 fluorophore. PI is excited by a 488 nm laser and detected by a filter corresponding to detection of PE fluorophore.
  APC-conjugated annexin V is excited by a 640 nm laser and detected by a filter corresponding to detection of APC fluorophore. Acquire 10000 events.
- *Template 3:* for BrdU/PI staining:
  PI is excited by a 488 nm laser and detected by a filter corresponding to detection of PE fluorophore. Alexa Fluor 647-conjugated antibody targeting BrdU is excited by a 640 nm laser and detected by a filter corresponding to detection of APC fluorophore. Acquire 10000 events.

For each template, initially gate the primary cell population using forward scatter area (FSC-A) and side scatter area (SSC-A) to identify cells based on size and granularity. Subsequently, apply FSC-A and FSC-H gating to exclude doublets, ensuring that only single cells are analyzed. Finally, using stained samples, adjust PMT voltages. If using multiple different cell lines, find optimal SSC-A and FSC-A values suitable for analysis of different cell lines within the same template. For simple samples represented by one cell line, the acquisition of 10000 events provides enough data and minimizes the acquisition time, which decreases the gap between the first and the last samples measured and, therefore, increases the overall homogeneity of the analysis. This protocol is easily adaptable. Users can modify the combinations of antibody-fluorophores, detection channels, and excitation lasers based on the flow cytometers available to them.

#### Additional preparations for cell cycle analysis

2 N HCl/0.5% Triton X-100 Solution:

1. Prepare this solution by mixing one part Triton X-100 stock solution with 199 parts of 2 N HCl.
2. Store the solution at room temperature.

Borax anhydrous - sodium tetraborate:

1. To prepare 300 ml of 100 mM sodium tetraborate at pH 8.5, dissolve 6 g of sodium tetraborate in 300 ml of deionized water (ddH_2_O).
2. Adjust the pH to 8.5 using a pH meter and hydrochloric acid.
3. Filter the solution through a 0.45 µm filter.
4. Store the prepared solution at room temperature.

RNase A preparation:

1. To prepare 1 ml of RNase A solution (10 mg/ml), dissolve 10 mg of RNase A in 1 ml of sterile Dulbecco’s Phosphate Buffered Saline (DPBS).
2. Aliquot the solution and store at −20 °C to maintain enzyme activity.

DNA staining mix:

1. For DNA staining, prepare a mix containing 0.5% Tween 20 in DPBS, along with anti-BrdU antibody, PI, and RNase A.
2. For 10 samples (500 µl total, 50 µl per sample), mix the following: 2.5 µl of 100% Tween-20, 486 µl of DPBS, 10 µl of anti-BrdU antibody, 1 µl of PI (to achieve a final concentration of 3 µM), and 0.5 µl of RNase A (to achieve a final concentration of 10 µg/ml).
3. Always prepare this mix fresh to ensure optimal staining efficacy.

#### Additional preparations for measurement of cell death and apoptosis

Annexin V binding buffer:

1. Prepare a 10× concentrate of Annexin V binding buffer containing 0.1 M HEPES (pH 7.4), 1.4 M NaCl, and 25 mM CaCl_2_.
2. Filter the solution through a 0.45 µm filter.
3. Store the buffer at +4 °C.
4. Before use, prepare a 1× working solution by diluting the 10x concentrate 1:10 with ddH_2_O.

Annexin V/PI staining mix:

1. To prepare the annexin V/PI staining mix for 10 samples (500 µl total, 50 µl per sample): mix 50 µl of annexin V binding buffer with 429 µl of ddH_2_O, add 20 µl of Annexin V (2 µl per test) and 1 µl of PI to achieve a final concentration of 3 µM.
2. Always prepare this mix fresh to ensure optimal staining efficacy.
3. The fluorescence from CellTrace Violet, PI, and Annexin V-APC is excited by three separate lasers: CellTrace Violet is excited by a violet laser (400 nm), PI by a blue laser (488 nm) and Annexin V-APC by a red laser (640 nm). This multi-laser setup allows for the simultaneous acquisition of all three dyes in the same sample without the need for compensation.

#### Additional preparations for measurement of proliferation

CellTrace Violet staining solution:

1. To prepare a staining solution with a concentration of 3 µM CellTrace Violet, dilute the 2 mM stock solution of CellTrace Violet by a factor of 666.7 in DPBS.
2. Protect the solution from light until it is used.

#### Additional preparations for MMP measurement

JC-1 dye:

1. Prepare a 30 µM JC-1 staining solution by diluting the 7.66 mM stock solution by a factor of 1:255.3 in complete media.
2. Immediately after preparation, vigorously vortex or sonicate the solution to ensure complete dissolution of the dye. Note that 30 µM is not the final concentration used for staining the cells; this solution will be further diluted to a final concentration of 3 µM when mixed with the cells.

JC-1 dye compensation:

Proper compensation of the JC-1 fluorescent signal is crucial for obtaining accurate and relevant data on MMP. JC-1 dye can exist in two forms: as a monomer that emits green light when excited by a 488 nm blue laser, detected by a filter corresponding to FITC fluorescence, and as a J-aggregate that emits red light under the same excitation, detected by a filter corresponding to PE fluorescence [23]. Notably, the green-emitting monomers emit signals detected in both FITC and PE filters. This overlap results in significant spillover, with a substantial portion of the signal detected in the PE channel originating not from red J-aggregates but from green monomers. This leads to a situation where an increase in signal in one channel also increases the signal in the other channel. Consequently, the PE signal comprises two components: the spillover signal from monomers in the PE channel and the natural signal from J-aggregates.

**Fig. 3:**
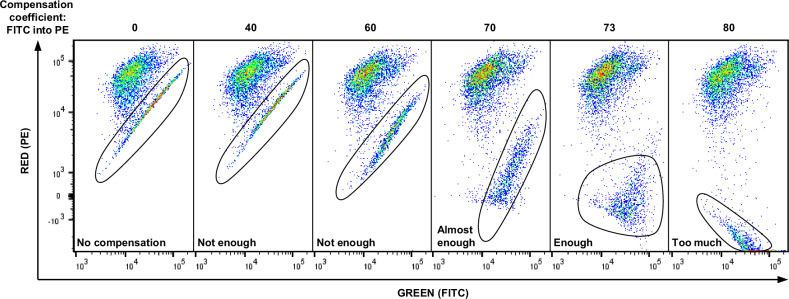
JC-1 compensation. The SW1417 cell line was stained with 3 µM JC-1 and analyzed using flow cytometry. Various compensation settings were applied to correct for spillover fluorescence from green-emitting monomers, which emit light detectable in both the FITC channel and the PE channel that captures the signal from red-emitting J-aggregates. Left graph: No compensation applied. This graph shows a diagonal-like distribution of fluorescence, indicating an incorrect assessment of the fluorescence signals where an increase in green fluorescence leads to a corresponding increase in red fluorescence, typical of unadjusted fluorescence spillover. Middle graphs: These graphs display various compensation settings, with the optimal compensation identified around a value of 73. At this setting, the distribution of fluorescence begins to resemble a normal cloud-like pattern, indicating effective separation of signals and minimal spillover effects. Right graph: Illustrates overcompensation. Here, the adjustment leads to an inverse diagonal pattern, where increases in one signal incorrectly correspond to decreases in the other, demonstrating the effects of excessive compensation adjustments.

Compensation is necessary to address this technical challenge. Typically, compensation involves preparing single-stained samples; however, due to JC-1’s dual emission properties from its two forms, preparing a single stain for this dye is not feasible. Therefore, the only solution to this issue is to manually compensate for the signal coming from green-emitting JC-1 monomers (in the FITC channel) into the red detection channel (PE). This adjustment can be performed by modifying the spillover values from the FITC channel into the PE channel in the flow cytometer’s software settings. Below, we demonstrate the process of manual spillover adjustment using SW1417 colorectal cancer cells (Fig. 3). It is important to note that the steps described are specific to the BD FACSLyric system and may differ with other flow cytometers. In brief, the signal obtained from cells should exhibit a uniform appearance, resembling a cloud of events that follow a normal distribution (with more events concentrated towards the center and fewer towards the edges), rather than showing a diagonal increase in signal. In the scenario depicted in the left graph where no compensation is applied, a diagonal-like distribution of cell populations is evident when the signal is visualized as a dot-plot with axes corresponding to green and red fluorescence detected by FITC and PE filters, respectively. This diagonal pattern indicates that an increase in the green signal linearly increases the signal in the red channel across a wide range of intensities, which is not a natural distribution but rather an acquisition artifact.

When compensatory coefficients of 40 or 60 are applied, this diagonal-like population begins to diverge from the main population and expand in proportions, yet it does not achieve a cloud-like distribution, indicating that the compensation is still unsatisfactory. A coefficient of 70 further broadens the distribution, beginning to resemble a more normal-looking population of cells; however, traces of the diagonal pattern persist, suggesting a need for finer adjustments. A coefficient of 73 results in the population appearing as a cloud with a normal distribution at the edges, indicating that this level of compensation is acceptable (although it could potentially be refined further by reducing it to 72).

Importantly, a coefficient of 80 exemplifies overcompensation, where the distribution of cells again resembles a diagonal pattern, but in the opposite direction: an increase in signal in one channel corresponds to a decrease in another channel. This analysis demonstrates that optimal compensation exists within a narrow window but can be achieved with time and practice during pilot experiments.

#### Additional preparations for cell number measurements

Configure the flow cytometer to measure samples by time instead of counting events. Set the voltage of forward scatter (FSC) and side scatter (SSC) to accurately position the cells in the center of the dot plot. Gate only those events that correspond to cells with normal proportions. This involves excluding cell debris, which typically appears in the bottom left corner of a scatter plot, and apoptotic bodies, which are characterized by high SSC and low FSC values. In this study, we used the flow cytometer BD FACSLyric where 40 s of acquisition at the medium speed corresponds to 40 μl of sample acquired (1 μl/s). The accuracy of this quantification method was compared with a classic cell quantification technique using manual cell counting with a hemocytometer. To this end, 6×10^4^ of slow-proliferating D247MG cells and 4 × 10^4^ of fast-proliferating COLO-320 cells were seeded in 6-well plates and were allowed to continuously grow over a period of 4 days. Subsequently, cells were collected in 2 ml of media and the cell counts were assessed in 40 μl using the flow cytometer, resulting in 1.27 × 10^4^ cells (D247MG cell line) and 2.75 × 10^4^ cells (COLO-320). Comparison with hemocytometer counts yielded 1.39 × 10^4^ and 3.12 × 10^4^ cells respectively, showing deviations of only 9% and 13%, confirming the accuracy of the flow cytometry method.

The total number of cells calculated in the full volume of 2 ml was 0.635 million for D247MG and 1.375 million for COLO-320. This corresponds to fold changes in cell number of 10.56 and 34.375 respectively over the course of 4 days, highlighting the ability of this quantification technique to robustly detect the differences between slow- and fast-proliferating cell lines.

If your flow cytometer is not well-calibrated, maintained, or lacks precision in counting events and fails to produce reproducible measurements of cell counts, consider using flow cytometry counting beads [27]. Various options are available on the market. These beads facilitate the calculation of cell numbers in a sample by comparing the number of acquired beads, which are provided at a known concentration, to the number of cells. This method provides a reliable and accurate means to ensure the accuracy of cell counts, especially when instrument performance may be suboptimal.

### Step-by-step method details

#### Standard laboratory procedures

##### Centrifugation

All centrifugation steps should be performed at 250 g for 5 min at room temperature, unless otherwise specified.

##### Incubation

All incubation steps are conducted in an incubator set to 37 °C, within a humidified atmosphere containing 5% CO_2_, unless otherwise noted.

##### Cell washing

This process includes several steps: adding DPBS or media to a tube containing cells, centrifuging the tube to pellet the cells, aspirating the supernatant above the cell pellet, and resuspending the cells in a specified solution. Throughout this protocol, references to a “cell washing” step will encompass all these actions, and only washing solutions will be specified (mainly DPBS or complete media containing fetal bovine serum and antibiotics). The volume added for washing should be the maximum amount that the tube can accommodate without risk of spillage.

#### Day 0

Harvesting and counting of cells:

1. For suspension cells, directly collect them into a 15-ml tube. For adherent cells, after trypsinization, neutralize the trypsin and collect the cells in a similar tube. Centrifuge to pellet the cells and aspirate the supernatant.
2. Wash the cells in DPBS.
3. Resuspend the cell pellet in 1 ml of complete media. Count the cells using a hemocytometer or any other appropriate cell counting instrumentation available.

Pre-staining of cells with CellTrace Violet dye:

1. Determine the necessary number of cells and 6-well plates required. For a 6-well plate, plan for 4 × 10^4^ cells per well for fast-proliferating cells and 6 × 10^4^ cells per well for slow-proliferating cells. Designate one well per cell line for the D1 (day 1) staining control and another well per cell line for untreated cells (or control treatments like DMSO or scramble) to be measured on D4 (day 4). Additionally, allocate wells equal to the number of treatments (× wells) for the various treatment groups. To account for potential losses during sample preparation and centrifugation, increase your estimated cell requirement by 20%.
2. Transfer the calculated number of cells to a new 15-ml tube and wash with DPBS.
3. Resuspend the cells in a 3 µM CellTrace Violet dye staining solution. We recommend using 1–2 × 10^6^ cells per 2 ml of staining solution to ensure adequate staining.
4. Incubate the cells for 20 min at RT (room temperature, approximately 23 °C), ensuring they are protected from light.
5. After the incubation, add five volumes of complete media to dilute the original staining volume and incubate for an additional 5 min at RT.
6. Centrifuge the cells and aspirate the supernatant.
7. Wash the cells with complete media.
8. Resuspend the cells in the appropriate volume of complete media, aiming for a density of either 4×10^4^ or 6×10^4^ cells in 1.5 ml per well of a 6-well plate, depending on the proliferation rate of the cell lines.
9. Seed the cells in 6-well plates. Ensure that the D1 staining control cells are seeded on a separate 6-well plate. Place the seeded cells in an incubator.

#### Day 1

Measurement of D1 staining control cells and cell treatments:

1. Aspirate the media and wash the wells containing D1 control cells with 2 ml of DPBS. Aspirate the DPBS and add 500 µl of trypsin to each well. Allow the trypsin to act for 2–5 min to detach the cells.
2. Neutralize the trypsin by adding 1000 µl of complete media to each well. Transfer the cell suspensions from each well into a corresponding 1.5-ml tube for centrifugation.
3. Centrifuge the cells and aspirate the supernatant.
4. Wash the cells in DPBS.
5. Resuspend the cells in 200 µl of complete media.
6. Analyze the cell fluorescence using a flow cytometer set to detect the signal using the BV421 fluorescence channel in *template 2*.
7. Add the planned treatments to the corresponding wells of the experimental plates that contain cells to be assessed after 72 h.

#### Day 4

Addition of BrdU and cell collection:

**Fig. 4:**
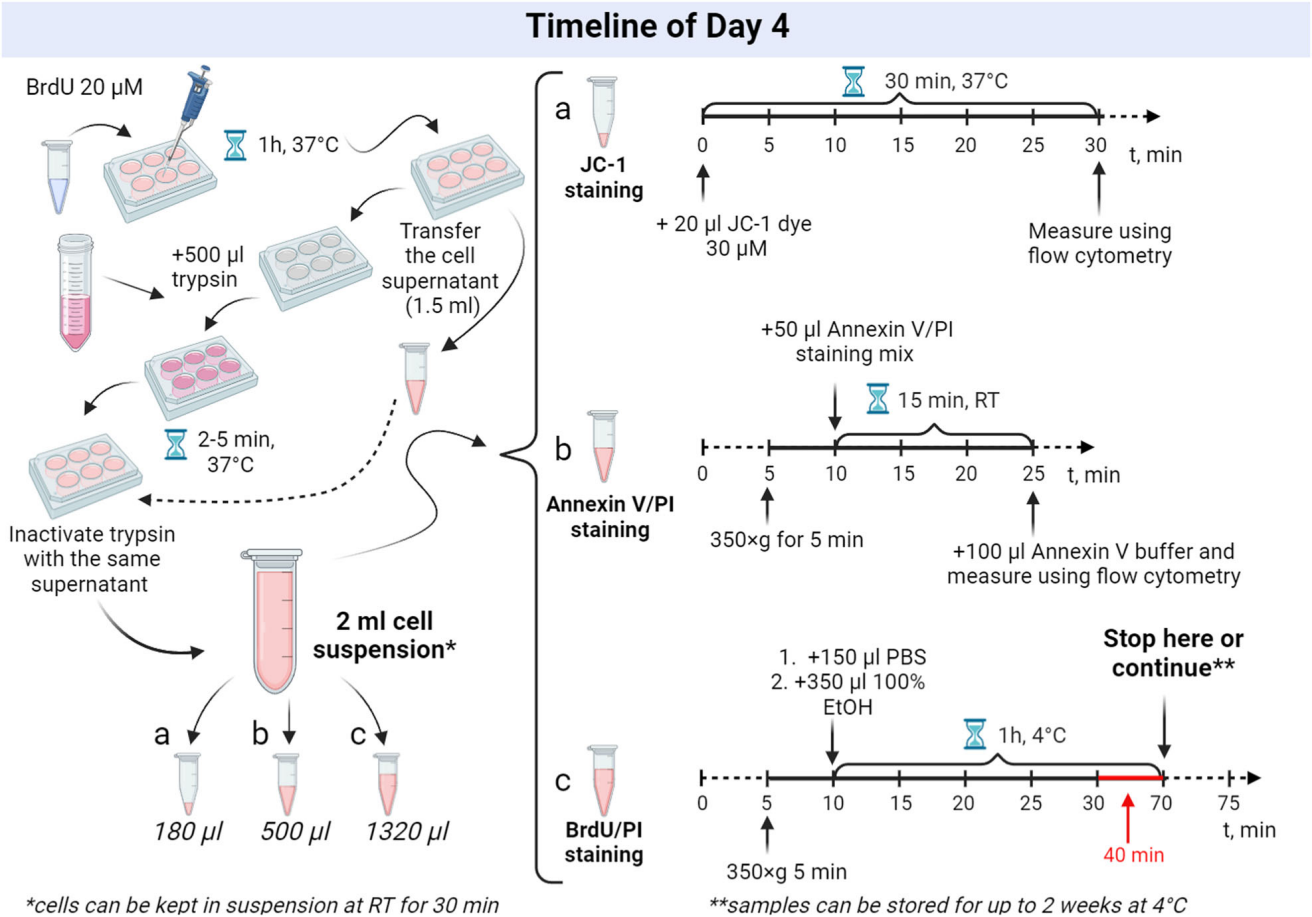
Timeline for day 4. Left part: Experimental cells are treated with BrdU and collected after a 1-h incubation. The cells are harvested using trypsinization, homogenized, and then transferred directly from each corresponding well into three separate 1.5-ml tubes for three independent stainings. Importantly, the trypsin is neutralized using the original cell supernatant collected from the corresponding cells. Right part: The obtained samples, containing 180 µl, 500 µl, and 1320 µl (or less) of cell suspensions, are utilized for three different stainings: JC-1 staining with cell counting, annexin V/PI staining, and BrdU/PI staining, respectively. Detailed protocols for each staining are provided in the text.

1. After incubating the treated and control cells for 71 h, 1 h before the endpoint of the experiment, add BrdU from a 20 mM stock solution directly to the cells to achieve a final concentration of 20 µM, without changing the media (Fig. 4).
2. Incubate cells for 1 h at 37 °C in an incubator (duration might vary depending on a cell line).
3. After the BrdU incubation, carefully transfer the cell supernatant, approximately 1.5 ml from each cell sample, into a separate clean 1.5-ml tube. Do not aspirate the cell media during this step, as detached and dying/apoptotic cells may be present in the supernatant. Similarly, avoid washing the wells with DPBS, as this could result in loss of weakly attached cells.
4. Add 500 µl of trypsin directly to the cells remaining in the wells and incubate for 2–5 min at 37 °C in an incubator (0.25% Trypsin-EDTA can efficiently act even if cells were not washed with DPBS and a smal amount of FBS-cointaing media is still present).
5. Use the previously collected supernatant to inactivate the trypsin directly in the 6-well plate, using the supernatant from the corresponding wells.
6. Thoroughly mix the cells in each well to ensure a homogeneous final solution. The resulting volume of cell suspension (approximately 2 ml) should be divided for different stainings as follows:
  *180* *µl for JC-1 staining;*
  *500* *µl for annexin V/PI staining;*
  *1320* *µl (or less) for BrdU/PI analysis*
7. Begin the staining process by adding JC-1 dye to the allocated 180 µl of cell suspension. While the cells are incubating with JC-1 dye, continue with annexin V/PI and BrdU/PI stainings. This parallel processing maximizes efficiency and reduces total protocol time.

**Assessment of MMP and cell number (timing:** ≈ **1** **h):**

1. Stain 180 µl of cell suspension by adding 20 µl of previously pre-diluted JС-1 dye (30 µM). The final concentration of JC-1 is 3 µM.
2. Incubate the cells at 37 °C in an incubator for 30 min.
3. After incubation, analyze the fluorescence signal using a flow cytometer with *template 1* specifically set up for JC-1 analysis. Record the number of cells detected over a 40-s acquisition period. Thoroughly vortex each sample before the acquisition. Store the samples in thermostat at 37 °C before analysis. Keep protected from light.

**Assessment of cells proliferation, apoptosis and viability (timing:** ≈ **1** **h):**

1. Centrifuge 500 µl of the cell suspension in a 1.5-ml tube at RT, 350 × *g* for 5 min.
2. Aspirate the supernatant and resuspend the cell pellet in 50 µl of annexin V/PI staining mix and incubate for 15 min at RT protected from light.
3. After incubation, add 100 µl of 1× annexin V binding buffer and analyze using a flow cytometer and *template 2*. Store the samples on ice before the flow cytometer analysis. Keep protected from light. Vortex before the sample acquisition.

**DNA synthesis analysis (timing:** ≈ **3** **h):**

1. Centrifuge the tube containing the remaining initial cell suspension at RT, 350×g for 5 min. Aspirate the supernatant.
2. For cell fixation and permeabilization first resuspend the cell pellet in 150 μl of DPBS and then slowly add 350 μl of ice-cold absolute EtOH. Mix gently and incubate at +4 °C for at least 1 h. After permeabilization and fixation samples can be stored for up to 2 weeks at 4 °C.
3. Ensure that the total number of permeabilized cells is between 3×10^5^-1×10^6^ cells per sample to ensure quality staining. Adjust the cell count based on data obtained from the JC-1 staining and cell counting. Discard any excess cell suspension if necessary.
4. Pellet the fixed cells by centrifugation at RT, 1000 × *g* for 5 min. Aspirate the supernatant.
5. Resuspend the cells in 200 μl of 2 N HCl/0.5% Triton X-100 and incubate for 20 min at RT.
6. Centrifuge the cells at RT, 1000 × *g* for 5 min and remove the supernatant.
7. Resuspend the cells in 200 μl of 0.1 M sodium tetraborate pH 8.5 and incubate for 2 min at RT.
8. Centrifuge the cells at RT, 1000 × *g* for 5 min and remove the supernatant.
9. Resuspend the cells in 1.5 mL of DPBS containing 2% FBS and centrifuge at RT, 1000 × *g* for 5 min.
10. Aspirate the supernatant and resuspend the cells in 50 μl of BrdU DNA staining mix. Incubate for 30 min at RT protected from light.
11. Add 150 μl of DPBS containing 2% FBS to the cells. Prepare the samples for analysis using a flow cytometer with *template 3* designated for BrdU/PI staining. Vortex before the sample acquisition. Keep protected from light.

### Expected results

In this study, we employed two colorectal cancer (CRC) and two glioma cell lines, along with four different treatments, to illustrate the potential outcomes achievable using the proposed methodology. However, this protocol is versatile and can be applied to any types of adherent and suspension cells, as well as various treatments.

SW480 and COLO-320 colorectal cell lines as well as LN428 and D247MG glioma cell lines were cultured in DMEM media supplemented with 10% fetal bovine serum and 1% penicillin/streptomycin at 37 °C in a humid atmosphere containing 5% CO_2_. Cell passaging was performed using a 0.25% trypsin-EDTA solution.

For the experiment, the cell lines were trypsinized and collected from 75 cm² flasks. Subsequently, the protocol described herein was implemented. In brief, after CellTrace Violet staining, SW480, COLO-320, and LN428 cell lines were seeded in 6-well plates at 4 × 10^4^ cells per well, while the D247MG cell line was seeded in 6-well plates at 6 × 10^4^ cells per well. The following day, baseline CellTrace Violet fluorescence measurements were taken from control cells of each cell line.

Treatment protocols were as follows: CRC cell lines received rotenone (200 nM) and antimycin A (200 nM) to inhibit mitochondrial electron transport chain (ETC) Complexes I and III, respectively. Meanwhile, glioma cell lines were treated with the chemotherapeutic agents temozolomide and carmustine, both at 20 µM concentrations, which primarily function through DNA alkylation and are commonly utilized clinically for glioma treatment. Following the 71-h incubation time, all experimental cells were treated with BrdU and incubated for another hour. Next, the cells were trypsinized and trypsin was inactivated using the media collected directly from the corresponding wells. Finally, obtained cell suspensions were divided into three tubes and were processed independently for (1) JC-1 staining and cell counting, (2) analysis of CellTrace Violet staining and annexin V/PI staining and (3) BrdU/PI staining.

#### Cell proliferation and cell counts

**Fig. 5:**
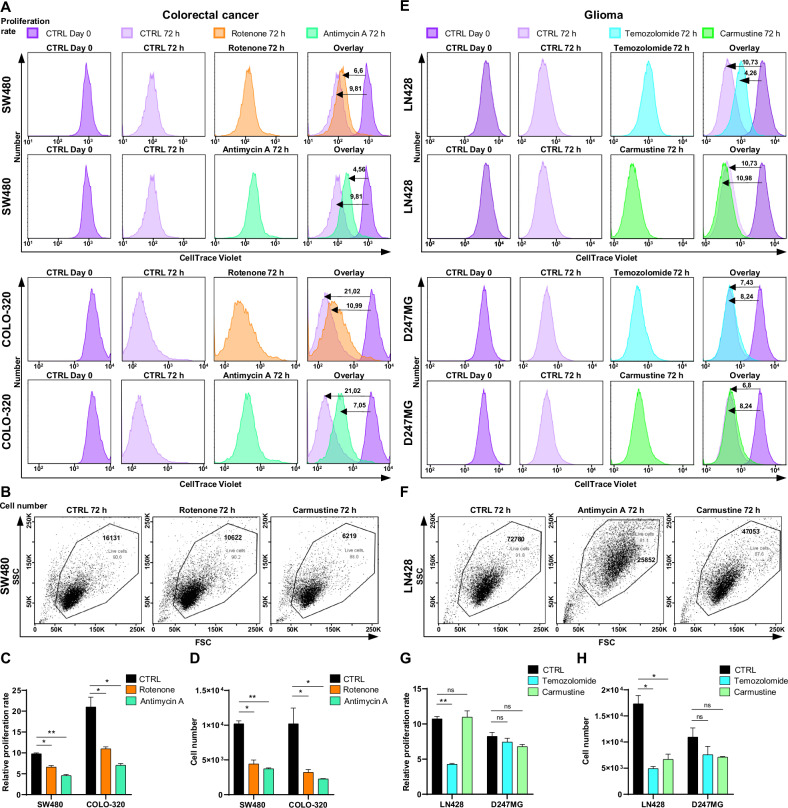
Analysis of cell number and proliferation rate changes in CRC and glioma cell lines after 72 h of treatment. **A** Representative CellTrace Violet histograms for SW480 and COLO-320 cell lines at day 1 (CTRL) and after 72 h, with and without 200 nM rotenone or 200 nM antimycin A treatment. **B** Flow cytometry dot plots of SW480 cells after 72 h, with and without 200 nM rotenone or 200 nM antimycin A treatment. **C** Relative proliferation rates of SW480 and COLO-320 cell lines with or without treatments, determined by CellTrace Violet staining. **D** Changes in absolute cell numbers measured by flow cytometry following rotenone and antimycin A treatments. **E** Representative CellTrace Violet histograms for LN428 and D247MG cell lines at day 1 (CTRL) and after 72 h, with and without 20 μM temozolomide or 20 μM carmustine treatment. **F** Flow cytometry dot plots of LN428 cells after 72 h, with and without 20 μM temozolomide or 20 μM carmustine treatment. **G** Relative proliferation rates of LN428 and D247MG cell lines with or without treatments, determined by CellTrace Violet staining. **H** Changes in absolute cell numbers measured by flow cytometry following temozolomide and carmustine treatments. All values are means ± SEM; “ns” indicates non-significant; *p < 0.05 and **p < 0.01; *n* = 3.

The proliferation rates of the cell lines were assessed using *Template 2* and results from CellTrace Violet staining. To determine the relative proliferation rate, the fluorescence intensity of CellTrace Violet on Day 1 was divided by that on Day 4. The analysis revealed a significant decrease in the relative proliferation rate of SW480 cells after 72 h of treatment with 200 nM rotenone, as compared to untreated controls (Fig. 5A, C). Moreover, treatment with 200 nM antimycin A resulted in an even greater reduction in the proliferation rate of SW480 cells, indicating greater sensitivity to antimycin A compared to rotenone. In contrast, the COLO-320 cell line initially exhibited a faster proliferation rate than the SW480 line, with a mean value of 21.02. However, this rate significantly dropped to 10.99 following rotenone treatment and further to 7.05 after exposure to antimycin A, demonstrating substantial sensitivity to these treatments.

Flow cytometry-based cell counting, performed using *Template 1* in conjunction with JC-1 staining, revealed significant reductions in cell numbers correlating with decreased proliferation rates following treatment with the compounds under study (Fig. 5B, D). Specifically, the SW480 cell line exhibited a 2.3-fold decrease in cell numbers following rotenone treatment and a 2.7-fold decrease following antimycin A treatment. Conversely, the COLO-320 cell line demonstrated greater sensitivity, with cell numbers decreasing by 3.2-fold under rotenone treatment and 4.5-fold with antimycin A, underscoring the higher susceptibility of the COLO-320 line to these interventions compared to the SW480 line.

The analysis of glioma cell proliferation demonstrated that the relative proliferation rate of the LN428 cell line decreased significantly from 10.73 to 4.26 after 72 h of treatment with 20 μM temozolomide, compared to untreated cells (Fig. 5E, G). However, treatment with 20 μM carmustine did not significantly alter the proliferation rate compared with that of the control. Conversely, the D247MG cell line exhibited a marginal decrease in proliferation rate from 8.24 to 7.43 following temozolomide exposure, and to 6.80 with carmustine treatment, though these changes were not statistically significant. The corresponding changes in absolute cell numbers aligned with the proliferation rates (Fig. 5F, H). Interestingly, temozolomide and carmustine did not affect the cell numbers in the D247MG line, suggesting its resistance to these types of treatments. In contrast, the LN428 line experienced a notable reduction in cell numbers, decreasing 3.5-fold with temozolomide treatment and 2.6-fold with carmustine. These data underscores that the D247MG cell line exhibits resistance to treatments commonly used for glioma.

#### Cell cycle analysis

**Fig. 6:**
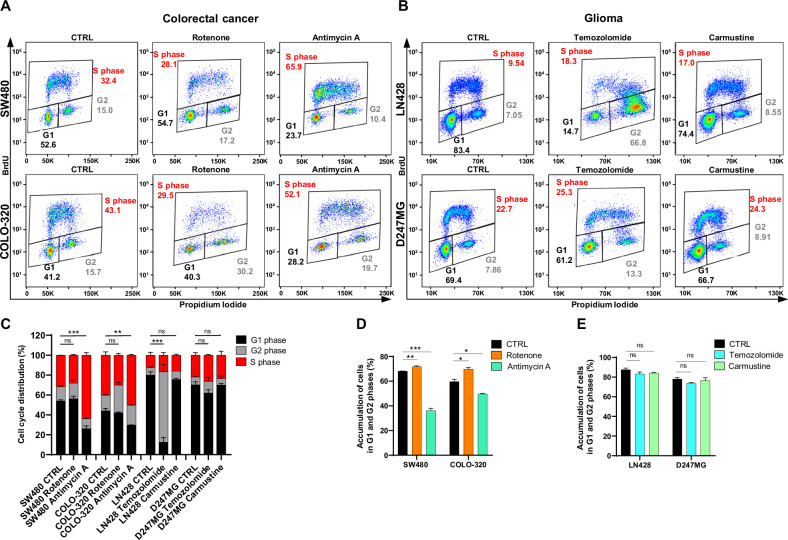
Analysis of cell cycle dynamics in CRC and glioma cell lines after 72 h of treatment. **A** Representative dot plots illustrating cell cycle frequencies of SW480 and COLO-320 cell lines after 72 h of treatment with and without 200 nM rotenone or 200 nM antimycin A, analyzed using BrdU/PI staining. **B** Representative dot plots depicting cell cycle frequencies of LN428 and D247MG cell lines after 72 hs of treatment with and without 20 μM temozolomide or 20 μM carmustine, analyzed using BrdU/PI staining. **C** Distribution of cells in G1, S, and G2 phases in control and treated CRC and glioma cell lines after 72 h of treatment. **D** Changes in the proportion of cells in G1 and G2 phases for SW480 and COLO-320 cell lines after treatment with 200 nM rotenone or 200 nM antimycin A, relative to untreated cells. **E** Changes in the proportion of cells in G1 and G2 phases for LN428 and D247MG cell lines after treatment with 20 μM temozolomide or 20 μM carmustine, relative to untreated cells. All values are means ± SEM; “ns” indicates non-significant; *p < 0.05, **p < 0.01 and ***p < 0.001; *n* = 3.

The cell cycle profiling conducted by using BrdU/PI staining and *template 3* characterized the distribution of G1, S, and G2 phases in CRC and glioma cell lines, both treated and untreated (Fig. 6). Dot plots elucidated the segregation of cells into three distinct populations across these phases for CRC cell lines (Fig. 6A) and glioma cell lines (Fig. 6B). For enhanced clarity, a histogram depicting the frequency of cells in each phase is presented in Fig. 6C. In the SW480 cell line, no statistically significant differences were observed in the G1 phase percentages between control and rotenone-treated cells. However, antimycin A treatment resulted in a significant reduction in the G1 phase proportion by 2.07-fold. Notably, a substantial number of cells transitioned to the S phase. Similarly, the COLO-320 cell line exhibited no change in the G1 phase after rotenone treatment compared to control. However, treatment with antimycin A led to a 1.5-fold reduction in the percentages of cells in both the G1 and G2 phases, implying a shift towards the S phase (Fig. 6D). These results indicate that antimycin A markedly diminishes the cell populations in G1 and G2 phases, augmenting the proportion of cells in the S phase. Conversely, the D247MG cell line displayed no significant alterations in cell cycle dynamics following treatments with temozolomide or carmustine, aligning with the unchanged proliferation rate and cell number count observed. Treatment of the LN428 cell line with temozolomide resulted in a dramatic 6.4-fold decrease in the percentage of cells in the G1 phase. Such an effect was absent with carmustine treatment, although the overall accumulation of cells in the G1 and G2 phases was comparable for both treatments (Fig. 6E).

#### Quantification of apoptotic and dead cells

Quantification of apoptotic and dead cells was performed using annexin V/PI staining and *template 2*. This staining technique facilitates the simultaneous detection of PI and annexin V, enabling the stratification and visualization of cells into four groups (Q1–Q4): Q1 represents early apoptotic cells (PI−/annexin V+), Q2 includes cells with permeable cytoplasmic membranes in the final stages of apoptosis (PI+/annexin V+), Q3 comprises cells predominantly undergoing non-apoptotic forms of death such as necrosis (PI+/annexin V−), and Q4 denotes healthy cells (PI−/annexin V−).

**Fig. 7:**
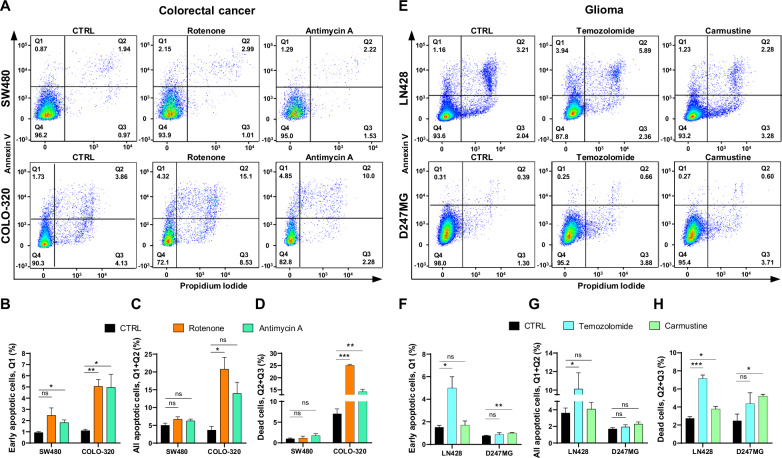
Quantification of apoptotic and dead cells in CRC and glioma cell lines after 72 h of treatment. **A** Representative dot plots depicting early apoptotic cells (Q1), late apoptotic cells undergoing apoptosis (Q2), cells dying without apoptosis induction (Q3), and live cells with no signs of apoptosis induction (Q4) in SW480 and COLO-320 cell lines after 72 h of treatment with and without 200 nM rotenone or 200 nM antimycin A. Detection of apoptotic and dead (permeable) cells was performed using annexin V/PI staining. **B–D** Changes in the numbers of early apoptotic cells, total apoptotic cells, and dead cells, respectively, following rotenone and antimycin A treatments compared to untreated cells. **E** Representative dot plots depicting early apoptotic cells (Q1), late apoptotic cells undergoing apoptosis (Q2), cells dying without apoptosis induction (Q3), and live cells with no signs of apoptosis induction (Q4) in LN428 and D247MG cell lines after 72 h of treatment with and without 20 μM temozolomide or 20 μM carmustine. Detection of apoptotic and dead (permeable) cells was performed using annexin V/PI staining. **F**–**H** Changes in the numbers of early apoptotic cells, total apoptotic cells, and dead cells, respectively, following temozolomide and carmustine treatments compared to untreated cells. All values are means ± SEM; “ns” indicates non-significant; *p < 0.05, **p < 0.01 and ***p < 0.001; *n* = 3.

In the case of the SW480 cell line, treatments with rotenone and antimycin A did not result in a significant increase in the number of dead (Q2 and Q3) and apoptotic cells (Q1 and Q2) after 72 h, suggesting that any increase might occur at earlier time points and may not be detectable at later stages (Fig. 7A–D). Conversely, the COLO-320 cell line exhibited an increased number of cells in these quadrants, potentially indicating a continued susceptibility to these treatments even at later stages.

The analysis of apoptosis and cell death in the LN428 and D247MG glioma cell lines revealed that temozolomide significantly increased the number of dead cells in both lines, whereas carmustine treatment led to an increase in dead cells only in the D247MG line (Fig. 7E–H). Early and total apoptosis assessments indicated that D247MG cells were not undergoing apoptosis after 72 h of treatment with temozolomide or carmustine. Therefore, the observed increase in dead cell counts may be attributed to the induction of alternative forms of cell death.

#### Evaluation of overall MMP and quantification of cells with high and low MMP

**Fig. 8:**
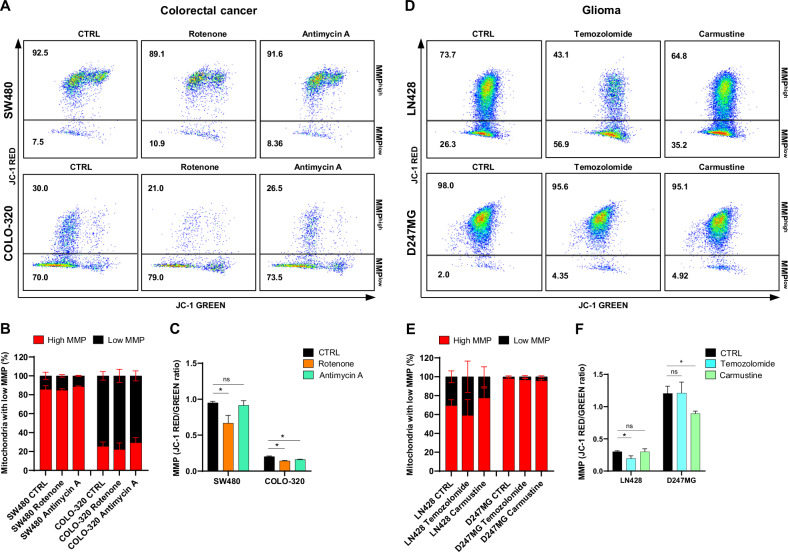
Analysis of mitochondrial membrane potential in CRC and glioma cell lines after 72 h of treatment. **A** Representative dot plots illustrating the distribution of cells with high MMP (JC-1 high RED signal) and low MMP (JC-1 low RED signal) in SW480 and COLO-320 cell lines after 72 h of treatment with and without 200 nM rotenone or 200 nM antimycin A. **B** The frequencies of cells characterized by high and low MMP in comparison between control and treated CRC cell lines after 72 h of treatment. **C** Changes in MMP among CRC cell lines after treatment with 200 nM rotenone or 200 nM antimycin A, relative to untreated cells. **D** Representative dot plots illustrating the distribution of cells with high MMP (JC-1 high RED signal) and low MMP (JC-1 low RED signal) in LN428 and D247MG cell lines after 72 h of treatment with and without 20 μM temozolomide or 20 μM carmustine. **E** The frequencies of cells characterized by high and low MMP in comparison between control and treated glioma cell lines after 72 h of treatment. **F** Changes in MMP among glioma cell lines after treatment with 20 μM temozolomide or 20 μM carmustine, relative to untreated cells. All values are means ± SEM; “ns” indicates non-significant; *p < 0.05, *n* = 3.

The analysis of MMP and quantification of cells with high and low MMP was performed using JC-1 staining and *template 1*. The analysis revealed major differences in mitochondrial membrane potential among the CRC cell lines (Fig. 8A). Specifically, the SW480 cell line displayed a high MMP in 85% of its cells, whereas the COLO-320 cell line predominantly exhibited low MMP, with only 25% of cells showing high MMP (Fig. 8B). Upon treatment with rotenone, a notable decrease in MMP was observed in both CRC cell lines. However, the impact of antimycin A was substantial only in the COLO-320 cell line (Fig. 8C).

In contrast, cells in the glioma cell lines, LN428 and D247MG, predominantly displayed high MMP, at 69% and 98% respectively (Fig. 8D-E). Interestingly, neither temozolomide nor carmustine significantly altered the MMP, but carmustine treatment significantly decreased the MMP of the D247MG cell line (Fig. 8F).

### Summary and bubble plot visualization

The data obtained from all conducted experiments is summarized in Table 4.

**Table 4:** Effects of mitochondrial inhibitors and chemotherapeutic agents on cell proliferation, cell cycle distribution, mitochondrial function, apoptosis, and cell death in CRC and glioma cell lines.

Cell line	SW480	SW480	SW480	COLO320	COLO320	COLO320	LN428	LN428	LN428	D247MG	D247MG	D247MG
Treatment	CTRL	Roten.	Antim.	CTRL	Roten.	Antim.	CTRL	Temoz.	Carm.	CTRL	Temoz.	Carm.
Cell number	10202 ± 616	4431 ± 799	3729 ± 187	10205 ± 3204	3218 ± 583	2273 ± 57	17,349 ± 2197	4927 ± 564	6697 ± 1407	10,952 ± 2496	7584 ± 2247	7110 ± 165
Relative proliferation rate (D1/D4)	9.81 ± 0.25	6.60 ± 0.54	4.56 ± 0.38	21.02 ± 3.33	10.99 ± 0.68	7.05 ± 0.57	10.73 ± 0.49	4.26 ± 0.18	10.98 ± 1.26	8.24 ± 0.97	7.43 ± 0.78	6.80 ± 0.42
Cells in G1 and G2 phases (%)	68.17 ± 0.06	71.90 ± 0.71	36.20 ± 2.55	59.70 ± 3.30	69.85 ± 2.05	49.65 ± 0.64	87.53 ± 2.71	83.18 ± 2.79	84.15 ± 1.06	78.04 ± 2.62	73.80 ± 0.85	76.50 ± 3.96
Cells with depolarized mitochondria (%)	14.13 ± 3.72	15.03 ± 1.38	11.27 ± 0.64	74.53 ± 4.60	77.90 ± 6.97	70.70 ± 5.24	30.60 ± 6.29	40.87 ± 16.65	22.43 ± 10.56	1.90 ± 0.92	3.28 ± 1.37	4.10 ± 1.08
Mitochondrial membrane potential (RED/GREEN)	0.95 ± 0.04	0.67 ± 0.15	0.92 ± 0.09	0.20 ± 0.02	0.14 ± 0.00	0.16 ± 0.01	0.30 ± 0.02	0.20 ± 0.06	0.30 ± 0.07	1.21 ± 0.19	1.21 ± 0.24	0.89 ± 0.06
Early apoptotic cells (%)	0.94 ± 0.21	2.47 ± 1.16	1.82 ± 0.42	1.10 ± 0.30	5.07 ± 1.52	4.97 ± 2.02	1.52 ± 0.29	5.00 ± 1.73	1.71 ± 0.64	0.78 ± 0.05	0.88 ± 0.27	1.01 ± 0.10
Total apoptotic cells (%)	5.02 ± 1.05	6.72 ± 1.22	6.26 ± 0.89	3.66 ± 1.48	20.75 ± 4.74	13.96 ± 5.47	3.60 ± 1.06	10.11 ± 3.03	4.07 ± 1.42	1.70 ± 0.31	1.93 ± 0.47	2.28 ± 0.45
Dead cells (%)	1.00 ± 0.41	1.14 ± 0.86	1.77 ± 1.06	7.03 ± 2.86	25.10 ± 0.99	14.25 ± 2.47	2.75 ± 0.49	7.15 ± 0.66	3.77 ± 0.51	2.48 ± 1.27	4.37 ± 2.09	5.22 ± 0.34

SW480 and COLO-320 (CRC cell lines), LN428 and D247MG (glioma cell lines) were treated with specific agents to assess their cellular responses. Treatments included rotenone (Roten.; 200 nM), and antimycin A (Antim.; 200 nM) for CRC cells, and temozolomide (Temoz.; 20 μM) and carmustine (Carm.; 20 μM) for glioma cells. After 72 h of incubation, various cellular parameters were measured, including: cell number (determined using flow cytometry-based cell counting), relative proliferation rate (calculated from CellTrace Violet fluorescence intensity ratios between Day 1 and Day 4), percentage of cells in G1 and G2 phases (assessed using BrdU/PI staining to determine cell cycle distribution), percentage of cells with depolarized mitochondria (measured using JC-1 staining to identify cells with low mitochondrial membrane potential) mitochondrial membrane potential (red/green ratio quantified using JC-1 fluorescence intensity ratios) percentages of early apoptotic and all apoptotic cells (identified using annexin V/PI staining to detect early and total apoptosis) and percentage of dead/dying cells (determined by the uptake of PI in annexin V/PI staining). Data are presented as mean ± SEM from three independent experiments.

The conducted analysis revealed several distinct characteristics regarding the drug response of the studied cell lines to the treatments administered. Notably, antimycin A, but not rotenone, increased the frequency of CRC cells in the S phase, leading to a significant reduction in the number of cells in the G1 and G2 phases. This effect was particularly pronounced in the SW480 cell line and was also clearly observable in the COLO-320 line.

An additional intriguing finding was that both temozolomide and carmustine significantly reduced the number of LN428 cells. However, only temozolomide effectivly in decreased the proliferation rate of these cells. The results of the cell cycle analysis supported this observation, showing that temozolomide, but not carmustine, induced cell cycle arrest in the G1 phase of the LN428 cell line.

This analysis exemplifies how different components of this workflow can complement each other and elucidate the biological mechanisms underlying changes in cell numbers following treatment. Furthermore, the relationship between apoptosis induction and mitochondrial depolarization was closely linked in the majority of treatments and cell lines, indicating that these two staining techniques provide a comprehensive overview of cell stress and apoptosis. For example, temozolomide significantly induced both mitochondrial depolarization and apoptosis in the LN428 cell line, in contrast to carmustine, which had a minimal impact on these parameters.

**Fig. 9:**
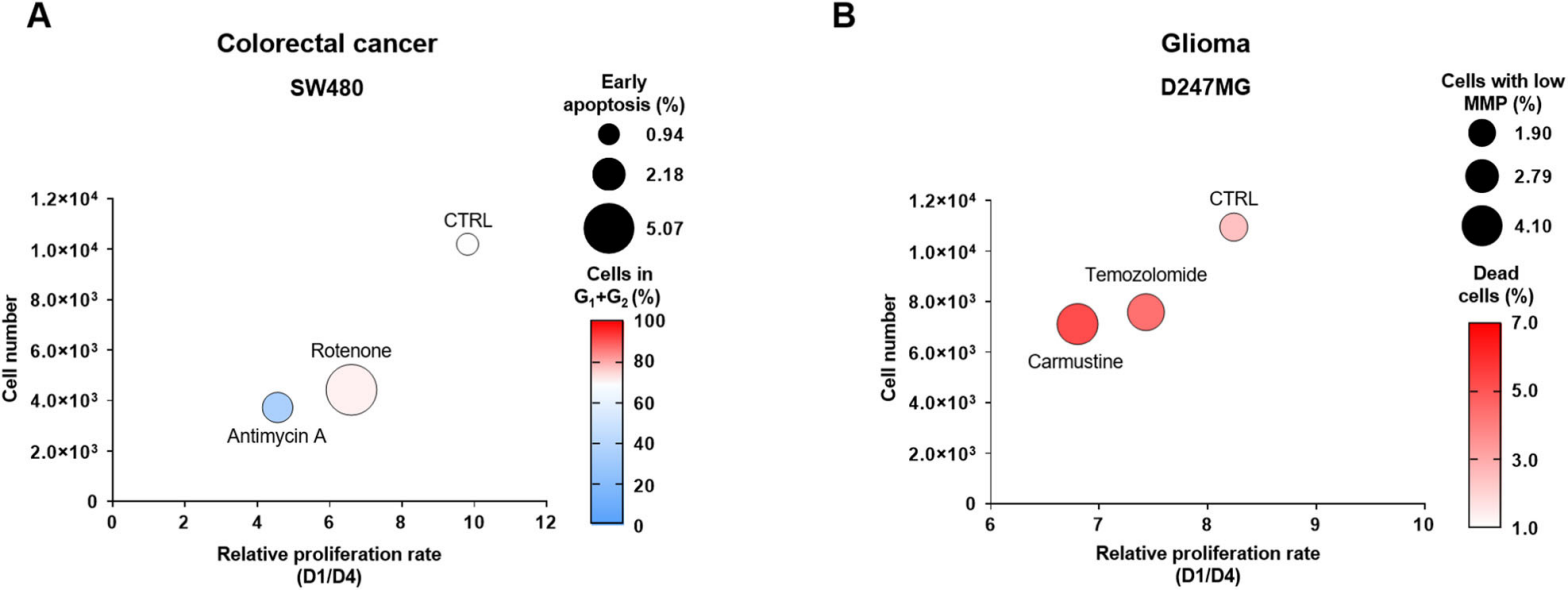
Analysis of compound effects induced by pharmacological treatments in SW480 and D247MG cell lines after 72 h. **A** Multiparametric analysis of SW480 cells treated with 200 nM rotenone or 200 nM antimycin A for 72 h, compared to control cells. The bubble plot integrates changes in cell number (flow cytometry cell counting, y-axis), early apoptosis levels (annexin V/PI staining, bubble size), proliferation rate (CellTrace Violet staining, x-axis), and cell cycle dynamics (BrdU/PI staining, bubble color; values represent the sum of cells in the G1 and G2 phases). **B** Multiparametric analysis of D247MG cells treated with 20 μM temozolomide or 20 μM carmustine for 72 h, compared to control cells. The bubble plot integrates changes in cell number (flow cytometry cell counting, y-axis), number of cells with low mitochondrial membrane potential (MMP; JC-1 staining, bubble size), proliferation rate (CellTrace Violet staining, x-axis), and number of dead cells (annexin V/PI staining, bubble color). All bubbles represent means; *n* = 3.

Furthermore, the results from these staining techniques can be effectively summarized and visualized through bubble plots that integrate four key parameters simultaneously. For example, a single bubble could encompass information regarding changes in cell numbers, proliferation rates, early apoptosis, and cell cycle dynamics (Fig. 9A). Specifically, the bubble plot for SW480 cells demonstrates that rotenone primarily reduces cell numbers through the induction of apoptosis and shifts the accumulation of cells towards the G1 and G2 phases, while antimycin A has a more pronounced effect on inhibiting proliferation and causing the accumulation of cells in S phase.

A second bubble plot illustrates that 20 μM carmustine is more effective against D247MG cells compared to 20 μM temozolomide. This is evident from consistent changes across four parameters: a decrease in cell number and proliferation rate, coupled with an increase in levels of mitochondrial depolarization and the number of dead cells (Fig. 9B). These bubble plot visualizations provide a clear and comprehensive overview of the complex interactions and effects of these treatments on the studied cell lines.

### Statistical analysis, software and visualizations

Data visualization was performed using GraphPad Prism 9.5.1 and FlowJo 10.9 software, the latter of which was also used for flow cytometry data analysis. Flow cytometry data were originally acquired using BD FACSuite™ software. Graphs display mean values obtained from *n* biological replicates, with error bars representing the standard error of the mean (SEM). An unpaired, two-tailed *t*-test was used for statistical analyses. Statistical significance levels are indicated as follows: *p* ≤ 0.05; *p* ≤ 0.01; *p* ≤ 0.001; *p*  ≤ 0.0001; “ns” indicates non-significant differences. Figures 1, 2, 4, and the graphical abstract were created using BioRender.com.

## Data Availability

The data that support the findings of this study are available in the Method section. Further inquiries can be directed to the corresponding author.
